# Development of an Indirect ELISA to Distinguish between Porcine Sapelovirus-Infected and -Vaccinated Animals Using the Viral Nonstructural Protein 3AB

**DOI:** 10.3390/cimb46090583

**Published:** 2024-09-03

**Authors:** Zuchang Zhong, Benqiang Li, Jie Tao, Jinghua Cheng, Ying Shi, Pan Tang, Jiajie Jiao, Huili Liu

**Affiliations:** 1 Institute of Animal Husbandry and Veterinary Medicine, Shanghai Academy of Agricultural Sciences, Shanghai 201106, China; zhongzuchang1@163.com (Z.Z.); huteng2010@saas.sh.cn (B.L.); taojie@saas.sh.cn (J.T.); zero5cheng@saas.sh.cn (J.C.); shiying@saas.sh.cn (Y.S.); tpvip2023@saas.sh.cn (P.T.); jiaojiajie@saas.sh.cn (J.J.); 2 National Demonstration Center for Experimental Fisheries Science Education, Shanghai Ocean University, Shanghai 201306, China; 3 Shanghai Key Laboratory of Agricultural Genetic Breeding, Shanghai 201106, China; 4 Shanghai Engineering Research Center of Pig Breeding, Shanghai 201302, China

**Keywords:** porcine sapelovirus, 3AB, indirect ELISA, infected and vaccinated animals

## Abstract

Porcine sapelovirus (PSV) is a new pathogen that negatively impacts the pig industry in China. Affected pigs experience severe diarrhea and even death. Vaccination is used to control disease outbreaks, and sensitive diagnostic methods that can distinguish infected animals from vaccinated animals (DIVA) are essential for monitoring the effectiveness of disease control programs. Tests based on the detection of the nonstructural protein (NSP) 3AB are reliable indicators of viral replication in infected and vaccinated animals. In this study, the recombinant PSV 3AB protein was expressed by a prokaryotic expression system, and an indirect ELISA method was established. Serum samples from healthy animals, immunized animals, and infected animals were evaluated. The ELISA method identified 3AB with high sensitivity (99.78%) and specificity (100.0%), and no cross-reaction was observed with serum antibodies against porcine reproductive and respiratory syndrome virus (PRRSV), infection with classical swine fever virus (CSFV), pseudorabies virus (PRV), bovine viral diarrhea virus (BVDV), porcine epidemic diarrhea virus (PEDV), or foot-and-mouth disease virus (FMDV). The ELISA method described here can effectively distinguish infected and vaccinated animals and is an important inexpensive tool for monitoring serum and controlling PSV.

## 1. Introduction

Porcine sapelovirus (PSV) is a nonenveloped single-stranded positive-sense RNA virus that belongs to the Sapelovirus genus of the Picornaviridae family [1]. PSV was first isolated from the intestines of pigs with diarrhea in the 1960s. To date, porcine PSV has been found in China [2], Brazil [3], South Korea [4], the United States [5], France [6] and India [7]. PSV is mainly transmitted through the fecal-oral route, leading to acute diarrhea, skin lesions, severe neurological disorders, and reproductive disorders in pigs [2,8,9,10].

The PSV genome has a length of approximately 7.5 kb, which is similar to that of other picornaviruses, and contains 5′ untranslated regions (UTRs), a single open reading frame (ORF), a short 3′ UTR, and a poly (A) tail [11]. The ORF encodes a single polyprotein, which is cleaved by viral proteases into one leader protein, four structural proteins (VP1-VP4), and seven nonstructural proteins (2A-2B-2C-3A-3B-3C-3D) [12]. The capsid protein VP1 is the most essential and highly variable viral protein in picornaviruses, and it serves as a crucial virulence factor [13,14]. An indirect ELISA method based on the PSV VP1 protein was developed, and this assay specifically detects antibodies induced by PSV infection and is helpful for preventing and controlling PSV [15].

FMDV infection can induce the production of antibodies against SPs and NSPs in animals, while vaccination can induce the production of high titers of SPs [16]. Thus, a method to detect NSP-specific antibodies is needed to distinguish between infected animals and vaccinated animals. In previous reports, the recombinant nonstructural proteins 3A [17,18], 3B [18,19], 3C [20], 3AB [21,22], 3ABC [23,24,25,26], and 3D [27] were used as coating antigens to establish an indirect ELISA detection method that can differentiate between FMDV-infected and vaccine-immunized animals. Among these proteins, the 3AB protein is the most specific and sensitive coating antigen [28]. As an emerging pathogen, PSV belongs to the same small RNA virus family as FMDV and possesses similar gene structures and characteristics. PSV infections often occur alongside other enterovirus infections and causes diarrhea. As PSV is neglected, this disease negatively affects the pig industry. Currently, no commercially available vaccine can prevent and control PSV infection, and distinguishing animals with PSV infections from vaccinated animals is an important goal.

In this study, a pET-28a-3AB recombinant plasmid was constructed by using a prokaryotic expression system to express the recombinant protein 3AB. Then, the 3AB protein was used as an antigen to establish an i-ELISA method that could distinguish between PSV-infected and vaccinated animals. The method can provide technical support for PSV prevention and control and has lain the foundation for the development and application of kits.

## 2. Materials and Methods

### 2.1. Serum Samples

(I) Sera from naïve pigs: Forty-six serum samples were collected from pigs with no virus infection or vaccination. The serum was collected from a pig farm in Shanghai, China, and no neutralizing antibodies were detected in these serum samples. (II) Sera from vaccinated pigs: Seventy-five serum samples were collected at 21–60 dpv from PSV-free pigs after secondary vaccination. Pigs were immunized with a PSV-inactivated vaccine at a PSV-free pig farm in Shanghai. (III) Sera from infected pigs: Sixty-two serum samples were collected at 5–14 dpv from infected pigs. PSV-positive sera were collected from pigs infected with PSV in a pig farm in Shanghai, and the neutralization test confirmed the PSV infection. (IV) Classical swine fever virus (CSFV)-positive serum was purchased from Beijing Kingbaio Biotechnology Co., Ltd. (Bejing, China), and bovine vial diarrhea virus (BVDV)-positive serum was purchased from Ingenasa in Spain; porcine epidemic diarrhea virus (PEDV)-, pseudorabies virus (PRV)-, foot-and-mouth disease virus (FMDV)-, and porcine reproductive and respiratory syndrome virus (PRRSV)-positive sera were stored in a laboratory.

### 2.2. Expression of Recombinant Protein

Viral RNA was extracted with TRIzol reagent. Primers were designed according to the SHCM2019 strain (GenBank: MN685785), and the target gene sequence was amplified from viral RNA by RT-PCR. The 366 bp (3AB) DNA fragment was cloned and inserted into the pET28a vector to generate the recombinant expression vector pET28a-3AB. The plasmid was transformed into *E. coli* BL21 (DE3)-competent cells. Single colonies were selected and inoculated into LB media supplemented with 50 μg/mL kanamycin for continuous culture. When the OD600 reached 0.6, isopropyl-β-D-1-thiogalactoside (IPTG) at a final concentration of 1 mM was added to induce expression for 6 h. Successful expression was confirmed by sodium dodecyl sulfate-polyacrylamide gel electrophoresis (SDS-PAGE) analysis.

### 2.3. Purification of Recombinant Protein

The bacterial pellet was resuspended in washing buffer (8 M urea, 50 mM Tris-HCl, 0.5 M NaCl, pH 8.0), sonicated for 10 min, and centrifuged to collect the supernatant. The supernatant was loaded directly onto a Ni2+-NTA column (equilibrated with washing buffer) and incubated at 4 °C for 2 h. The flow-through was collected, and the column was washed with washing buffer that contained 20 mM imidazole (with a volume 5 times the column volume). Protein elution was then performed using elution buffer containing 50 mM, 100 mM, 200 mM, and 400 mM imidazole. To visualize the expression and purification effect of the recombinant proteins, SDS-PAGE was performed. The purified recombinant protein was subjected to 20% SDS-PAGE and detected by Coomassie brilliant blue staining. Additionally, Western blotting was performed to identify the specific interaction between the recombinant protein and the primary antibody.

### 2.4. Development of the Indirect ELISA Protocol

The 3AB protein was diluted to different concentrations (2, 1, 0.5, and 0.25 μg/mL) using phosphate-buffered saline (PBS) as the coating solution. Each concentration was applied to two columns, and 50 μL of the coating solution was added to each well. The coating process was carried out at 37 °C for 2 h. Subsequently, the plate was washed five times with PBST (0.01 mol/L PBS containing 0.05% Tween-20, pH 7.4), with each wash lasting 3 min, and gentle tapping was performed to remove excess liquid. Then, 200 μL of blocking solution was added to each well and incubated at 37 °C for 1 h. After another round of washing and tapping, the negative and positive samples were diluted in PBS at concentrations of 1:400, 1:800, 1:1600, and 1:3200. The diluted samples, along with 100 μL per well, were added to the ELISA plate and incubated at 37 °C for 1 h. Following the same washing steps, 100 μL of HRP-labeled goat anti-pig IgG secondary antibody (diluted at 1:20,000) was added to each well and incubated at 37 °C for 30 min. The plate was then washed again and tapped dry. Next, 100 μL of TMB substrate solution was added to each well, and the plate was incubated at 37 °C in the absence of light for 10 min. The reaction was stopped by adding 100 μL of 2 mol/L H2SO4 to each well. The absorbance was read at a wavelength of 450 nm using an ELISA reader. The optimal antigen coating concentration and serum dilution were determined based on the antigen concentration and serum dilution of the well with the highest P/N value.

### 2.5. Determination of the ELISA Threshold Value

To determine the optimal threshold for the ELISA protocol, 45 serum samples were collected from clinically healthy animals with no history of foot-and-mouth disease. Additionally, 62 samples were obtained from uninfected animals that had received vaccination. Furthermore, 42 samples from infected animals that had not been vaccinated were tested. Receiver operating characteristic (ROC) curve analysis was performed using Graph Pad Prism 9 software to calculate the optimal cutoff value.

### 2.6. Positive Sample Sensitivity Assay

Three positive samples were taken and diluted in gradients of 1:100, 1:200, 1:400, 1:800, 1:1600, 1:3200, 1:6400, and 1:12,800. The detection results were analyzed for sensitivity.

### 2.7. Assay Reproducibility

Six different positive serum samples and six negative controls were used to assess the reproducibility of the assay. The serum of each animal was tested for three consecutive days using independently prepared ELISA plates. The average optical density (OD), standard deviation (SD), and coefficient of variation (CV) were calculated from the raw absorbance values.

### 2.8. Assay Specificity

The established method was used to detect CSFV-, PEDV-, PRV-, PRRSV-, FDMV-, BVDV-, and PSV-positive serum, as well as PSV-immunized serum. The specificity of the method was evaluated.

## 3. Results

### 3.1. Purification and Identification of the PSV 3AB Protein

**Figure 1:**
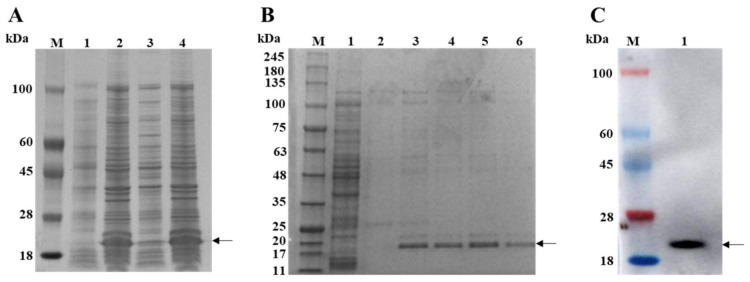
Purification and characterization of PSV 3AB. (**A**) Lane 1 is the pET-28a (+) control, lane 2 is the pET-28a-3AB after induction, lane 3 is the supernatant of the pET-28a-3AB after induction, and lane 4 is the inclusion bodies of the pET-28a-3AB after induction. (**B**) Lane 1 represents the flow-through liquid, lane 2 represents the washing process using washing buffer, and lanes 3–6 represent the 500 μL fractions collected sequentially using buffer containing 200 mM imidazole. (**C**) Western blot analysis of the recombinant 3AB protein. M is the protein ladder. The arrow indicates the location of the 3AB protein.

The expression of the recombinant bacterial solution of pET-28a-3AB was induced using IPTG. After sonication was performed to break the cells, the supernatant and pellet were collected separately for SDS-PAGE analysis. The results showed that the expressed protein was approximately 19 kDa in size, consistent with the predicted results, and the target protein was mainly present in the form of inclusion bodies (Figure 1A). The protein was dissolved in 8 M urea, and, after sonication, the supernatant was collected. The supernatant was purified by His affinity chromatography. The SDS-PAGE analysis showed that the target protein could be eluted after an elution buffer containing 200 mM imidazole was added (Figure 1B). The purified protein was desalted and concentrated using an ultrafiltration centrifuge tube. The protein concentration was determined to be approximately 726 µg/mL using a BCA protein assay kit. Western blot analysis using pig-derived polyclonal antibodies against 3AB showed specific recognition of the 3AB protein (Figure 1C).

### 3.2. Optimization of the Indirect ELISA Protocol

**Figure 2:**
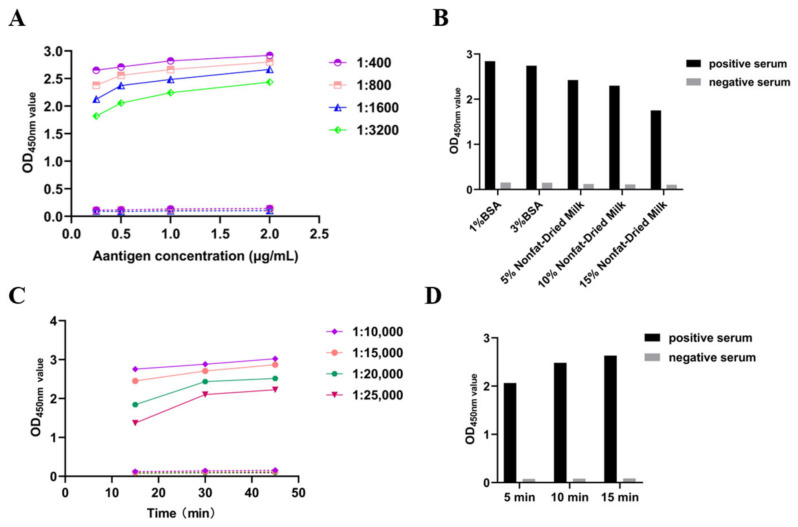
Optimization of the ELISA reaction conditions. (**A**) Screening of coating antigen concentration and serum dilution. (**B**) Screening of the blocking solution concentration. (**C**) Screening of the HRP-IgG dilution and incubation time. (**D**) Screening of the TMB reaction time.

To determine the optimal concentration of coating antigen (purified recombinant PSV 3AB protein) and dilution of test serum in an indirect ELISA, a checkerboard assay was performed (Figure 2). In addition, the optimal concentration of the blocking solution, incubation time, and dilution ratio of the secondary antibody, as well as the reaction time of TMB, were confirmed sequentially. After optimization, a 96-well ELISA plate was coated with protein at a concentration of 0.5 μg/mL in carbonate buffer and incubated at 37 °C for 2 h (Figure 2A). After a wash was performed with PBST, the plates were blocked with 10% nonfat dry milk at 37 °C for 2 h (Figure 2B). Then, PSV-positive serum and negative control serum (diluted 1:1600) were added to the plate and incubated at 37 °C for 1 h (Figure 2A). After another washing step, horseradish peroxidase-labeled goat anti-pig IgG (diluted 1:20,000) was added and incubated at 37 °C for 30 min (Figure 2C). Finally, 100 μL of TMB was added, and the mixture was incubated for 10 min to analyze antibody binding (Figure 2D).

### 3.3. Determining the Threshold Value of the Established ELISA

**Figure 3:**
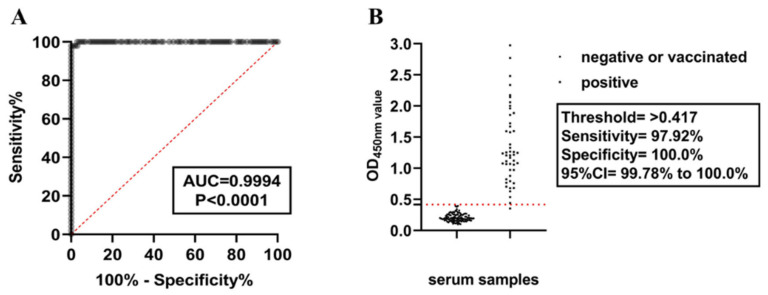
Diagnostic performance of the indoor ELISA was evaluated using serum from healthy, vaccinated, and infected animals. (**A**) ROC curve analysis was performed on the established 3AB ELISA with healthy (*n* = 41), vaccinated (*n* = 60), and infected (*n* = 50) serum. Healthy and vaccinated samples were used as uninfected controls. (**B**) According to the sensitivity, specificity, and 95% confidence interval (CI), the best threshold was determined by dot plot analysis.

The normal values of OD450nm in 149 known PSV status animal serum samples (41 negative samples, 60 vaccinated samples, and 48 positive samples) were determined using the ELISA method established in this study. The most appropriate threshold was determined through ROC curve analysis (Figure 3A). The area under the ROC curve (AUC) for the ELISA method established in this study was 0.9994 (95% CI = 0.9976~1.00), indicating that the ELISA method can distinguish between positive and negative sample groups. The optimal OD450nm value obtained through ROC curve analysis was 0.417 (Figure 3B), and diagnostic sensitivity (DSn) and diagnostic specificity (DSp) were further determined using a dot plot. The results showed that the DSn was 98.00% and the DSp was 100.0%, indicating that the ELISA method established in this study is suitable as a diagnostic test.

### 3.4. Evaluation of the Diagnostic Reproducibility, Specificity of the Established ELISA Method, and Positive Serum Sensitivity

**Figure 4:**
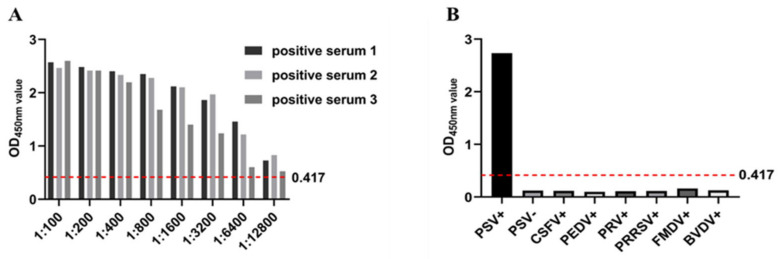
The diagnostic performance of the indoor ELISA was evaluated using serum from healthy, vaccinated, and infected animals. (**A**) ROC curve analysis was performed on the established 3AB ELISA with healthy (*n* = 41), vaccinated (*n* = 60), and infected (*n* = 50) serum. Healthy and vaccinated samples were used as uninfected controls. (**B**) According to the sensitivity, specificity, and 95% confidence interval (CI), the best threshold was determined by dot plot analysis.

To determine the optimal concentration of coating antigen (purified recombinant PSV 3AB protein) and dilution of test serum. The established ELISA method was used to compare positive sera and negative sera with different OD450nm values, and the intrabatch and interbatch variations on three different days were calculated to determine the detection reproducibility. The coefficient of variation was 0.22–4.61% for the intrabatch repeated test, and the coefficient of variation between batches was 1.03–7.38%. Both of these values were <10%, indicating that the method had good stability and high reproducibility (Table 1). Three PSV-positive serum samples were diluted eightfold, and an ELISA was carried out under the optimized conditions. The results are shown in the following table. When the serum was diluted 1:12,800, all three positive samples were positive, indicating that the method was highly sensitive (Figure 4A). CSFV-, PEDV-, PRV-, PRRSV-, FMDV-, BVDV-, and PSV-positive serum and negative control serum were detected. The results showed that only the PSV serum was positive, indicating that the test was specific and did not cross-react with other pathogens (Figure 4B).

### 3.5. Detection in Clinical Samples

The ELISA method was used to detect known serum samples, including 62 PSV-positive serum samples, 75 PSV-immune serum samples (16 serum samples collected on the 21st day after the second immunization, 19 serum samples collected on the 28th day after the second immunization, 37 serum samples collected on the 35th day after the second immunization, and 3 serum samples collected on the 60th day after the second immunization), and 46 negative serum samples (Table 2). The results showed that 61 of 62 positive serum samples were positive and 1 was negative, with a coincidence rate of 98.39%. Then, 75 negative serum samples were detected from 75 immunized pigs, and the coincidence rate was approximately 100.0%. Moreover, 46 negative samples were detected among the 46 negative samples, and the coincidence rate was 100.0%, indicating that the differential diagnosis ELISA established in this laboratory can effectively distinguish PSV-infected pigs from vaccinated pigs.

## 4. Discussion

In this study, the PSV nonstructural protein 3AB was expressed, prepared, and used as an antigen to establish an indirect ELISA method that can distinguish PSV vaccine-immunized animals from infected animals. The performance of this ELISA was evaluated using a series of sera collected from uninfected, infected, and vaccinated animals.

As early as the 1990s, researchers used foot-and-mouth disease virus, which belongs to the Picornaviridae family along with PSV, to establish ELISA methods for the differential animals infected with foot-and-mouth disease and vaccine-immunized animals [29,30]. Among them, the 3AB and 3ABC proteins had the greatest differential effects, and the sensitivity and specificity were as high as 94% [24,31]. There are currently many commercial ELISA kits on the market. In many areas worldwide, NSP ELISAs have become an integral part of vaccination-based control and monitoring programs. At present, there are reports of PSV in various countries worldwide, but PSV has only one serotype. In recent years, epidemiological investigations of PSV have revealed that the infection rate of PSV has increased in China [32]. To control the disease caused by PSV, an inactivated immune vaccine can be injected to achieve a preventive effect. Moreover, differential diagnosis and detection methods for immunized animals and infected animals are very important for preventing the abuse of vaccines, as well as preventing and controlling PSV. Therefore, according to the research and development of FMD detection methods, an ELISA method was established using the PSV 3AB protein for the differential diagnosis of PSV-infected and vaccine-immunized animals.

The PSV polyprotein 3AB was expressed in the form of inclusion bodies by an *E. coli* prokaryotic expression system. The hydrophilic N-terminus of the recombinant protein is essential for increasing the solubility of 3AB when expressed in *E. coli*. Therefore, the structural properties of 3AB were analyzed by the Hopp and Woods prediction methods, and the N-terminus of 3A exhibited weak hydrophilicity [21,33,34]. SDS-PAGE revealed that the size of the His-tagged 3AB protein was nearly 6 kDa, which was different from the calculated size (approximately 13 kDa) due to the His-tag size of the expression vector; however, the size had no effect on the target protein. In the study on FMDV, 3AB and 3ABC were the best antigens, and the established method and detected data were reliable [35]. The 3AB and 3ABC proteins are early products after translation, and the 3C protein exhibits protease hydrolysis activity and is responsible for cleavage during expression [36]. It has been reported that the expression of recombinant 3ABC in *E. coli* leads to a truncated protein similar to 3A [37]. To solve this problem, the 3C protein was mutated to reduce its enzymatic activity. Compared with the 3ABC protein, the 3AB protein is relatively simple to express and prepare, and site mutation treatment is not necessary. Previous studies have used baculovirus expression systems to express these proteins [38]. However, compared to bacterial systems, these methods are more expensive because they require cell culture and usually produce lower protein yields. The solubility of the PSV 3AB protein was poor, and 8 M urea was used to dissolve the inclusion body for denaturation and purification. Finally, the purified 3AB protein was desalted, concentrated in an ultrafiltration tube, and refolded into its natural structure; however, this method greatly reduced the efficiency of protein recovery. In future studies, the expression and purification steps will be further optimized to increase the protein yield per liter of culture medium to improve the recovery efficiency.

An indirect ELISA method for detecting antibodies in pig serum was established using the 3AB protein. Compared with competitive ELISA, which only targets one or two epitopes, indirect ELISA can be used, as the method possibly exhibits increased sensitivity due to the binding of multiple epitopes in the serum [25]. The purified 3AB protein had good reactivity, as detected by Western blotting. The optimized ELISA conditions showed that the optimal antigen concentration was 0.5 μg/mL, the optimal serum dilution was 1:1600, the optimal blocking solution was 10% skim milk powder, and the optimal working concentration of the enzyme-labeled secondary antibody was 1:20,000, followed by incubation at 37 °C for 30 min. The optimal critical value was determined to be 0.417 by receiver operating characteristic (ROC) curve analysis, and the diagnostic sensitivity and specificity were 97.92% and 100.0%, respectively. The method has good reproducibility, sensitivity, and specificity. This method was used to specifically test PSV and other serious viral positive serum samples, and found that there was no cross-reactivity. However, due to the limited number of sera and the absence of comparisons with the same small RNA viruses, we cannot completely exclude the possibility of cross-reactivity. Using the method to detect 183 known serum samples, 61 of 62 positive serum samples were correctly detected with a detection coincidence rate of 98.39%. Vaccination and healthy negative serum could be correctly detected, and the detection coincidence rate was 100%, indicating that this method could be applied to the differential diagnosis of PSV infection and vaccine-immunized animals. Unfortunately, as no commercial kit is available on the market, further comparisons cannot be made. The serum collected in this study was obtained from Shanghai with geographic limitations and a small number of serum samples. In the future, it will be necessary to expand the serum collection site and the number of serum samples for further optimization and improvement.

In conclusion, the PSV 3AB protein was expressed and purified by a prokaryotic expression system, and an indirect ELISA method was established for the differential diagnosis of PSV-infected and vaccine-immunized animals. Thus, this method provides a strategy for the detection and control of PSV and helps prevent vaccine abuse.

## Figures and Tables

**Table 1:** The established ELISA method for reproducibility evaluation. The test included 1 to 6 infected serum samples and 7 to 12 healthy or vaccinated serum samples. Mean = average. SD = standard deviation. CV = coefficient of variation.

Samples	Interbatch Variation		Intrabatch Variation	
	Mean + SD	CV (%)	Mean + SD	CV (%)
1	2.754 ± 0.014	0.51	2.732 ± 0.028	1.03
2	2.610 ± 0.006	0.22	2.639 ± 0.050	1.88
3	2.225 ± 0.039	1.75	2.240 ± 0.036	1.60
4	2.373 ± 0.050	2.10	2.388 ± 0.048	1.99
5	2.420 ± 0.043	1.76	2.430 ± 0.057	2.33
6	0.988 ± 0.011	1.10	1.025 ± 0.075	7.29
7	0.191 ± 0.009	4.61	0.189 ± 0.014	7.38
8	0.178 ± 0.004	1.98	0.167 ± 0.004	2.30
9	0.138 ± 0.002	1.20	0.128 ± 0.004	3.22
10	0.265 ± 0.003	1.13	0.272 ± 0.013	4.78
11	0.151 ± 0.005	3.28	0.152 ± 0.003	1.83
12	0.201 ± 0.002	1.06	0.200 ± 0.008	3.83

**Table 2:** The established ELISA method was used to detect 183 known serum samples.

	Samples	Positive	Negative	Concordance (%)
Healthy	46	0	46	100.0
Vaccinated	75	0	75	100.0
Infected	62	61	1	98.93

## Data Availability

The data are contained within the article.
